# A study on viruses and bacteria with particular interest on Mycoplasma pneumoniae in children with exacerbation of asthma from a tertiary care hospital in Sri Lanka

**DOI:** 10.1099/acmi.0.000778.v5

**Published:** 2024-07-30

**Authors:** Lakmini Inoka Wijesooriya, Victoria Chalker, Priyantha Perera, N. P. Sunil-Chandra

**Affiliations:** 1 Department of Medical Microbiology, Faculty of Medicine, University of Kelaniya, Colombo, Sri Lanka; 2 NHS Blood and Transplant, Bristol, UK; 3 Department of Peadiatrics, Faculty of Medicine, Wayamba University of Sri Lanka, Kurunegala, Sri Lanka

**Keywords:** exacerbation of asthma, macrolide resistance, MLST, *Mycoplasma pneumoniae*

## Abstract

Asthma is a significant public health concern, particularly in children with severe symptoms. Exacerbation of asthma (EOA) is life-threatening, and respiratory infections (RIs) play a crucial role. Though viruses play a significant role in EOA, patients are empirically treated with antibiotics, contributing to antibiotic resistance development. Although there are widely reported associations of EOA with viral or *Mycoplasma pneumoniae* infections, there are no published data for Sri Lanka. The present study aimed to identify the association of common respiratory viruses, typical respiratory bacterial pathogens and *M. pneumoniae* in children with EOA and relate them with the compatibility of antimicrobial use. A case-control study was conducted in the paediatric unit of North Colombo Teaching Hospital, Sri Lanka, involving two groups of children between 5 and 15 years of age. Group 1 is children with EOA and Group 2 is children with stable asthma (SA). Each group consisted of 100 children. Sputum/throat swabs were tested for common respiratory viruses using virus-specific fluorescein isothiocyanate-labelled monoclonal antibodies (MAbs), bacteria by routine culture, and *M. pneumoniae* by real-time polymerase chain reaction. Macrolide resistance in *M. pneumoniae* was detected using conventional PCR and sequencing specific genetic mutations in the 23S rRNA gene. * M. pneumoniae* was genotyped using nested multilocus sequence typing, which targeted eight housekeeping genes (*ppa*, *pgm*, *gyrB*, *gmk*, *glyA*, *atpA*, *arcC* and *adk*). There was no significant difference in age, gender, demographic or geographical location between the two groups. In children with EOA, antibiotics were used in 66 % (66/100) and macrolides in 42 % (42/100). Samples comprised 78 % (78/100) sputum and 22 % (22/100) throat swabs. Adenovirus was the most common virus identified, and it was significantly higher in children with EOA compared to those with SA. Still, the two groups had no significant difference in typical bacteria findings. *M. pneumoniae* was detected in one patient with EOA, but none was detected in the SA group. The *M. pneumoniae* was macrolide-sensitive and ST14 by multilocus sequence typing. This study showed that the empiric use of antibiotics in children with asthma might be better targeted with prior pathogen screening to inform appropriate treatment to minimize antibiotic resistance.

## Data Summary

All collected and analysed data from this work is reported in the article. Technical details of molecular detection of macrolide resistance and multilocus sequence typing of *Mycoplasma pneumoniae* are available at https://zenodo.org/doi/10.5281/zenodo.10574394 andhttps://zenodo.org/doi/10.5281/zenodo.10574437, respectively.

## Introduction

Asthma is a global and local healthcare burden that affects children and adults, substantially influencing day-to-day functions and leading to the absence of school attendance for children or work for adults. Per the World Health Organization, 262 million people in 2019 were affected by asthma globally [1]. The disease has a substantial negative impact on the health of children aged 10–14 years, the elderly aged 75–79 years and those who are immunocompromised.

Asthma is the 14th most crucial health disorder in the world concerning the extent and duration of disability [1]. In parallel, asthma has also become a worsening public health problem in Sri Lanka, with a population of about 22 million. According to Karunasekera *et al*., the prevalence of asthma among children (5–11 years of age) in Sri Lanka varies between 13 and 25 % [2]. This has been identified as a significant cause of school absence among primary school children in the country [3].

A considerable burden of the disease is mainly attributed to its exacerbation, defined as the interim worsening of symptoms of asthma provoked by an external stimulus [4]. The exacerbation of bronchial asthma (EOA) might be worsened, leading to life-threatening situations where, in some instances, the delay in hospital admission causes respiratory arrest. Asthma exacerbations are triggered by several factors primarily categorized as non-infective and infective causes. Non-infective causes may include dust particles, pollens, cigarette smoke, food allergies, different types of medicines or a combination of these factors, which likely vary in other individuals [5].

However, respiratory tract infections also play a more significant role in patients with EOA. It has been reported that viral or bacterial infections, including *Mycoplasma pneumoniae* or exposure to other agents (mites/fungal spores), were observed in 70 % (34/48) of inpatients with asthma exacerbation in clinical practice [6]. Viruses were identified in 14–49 % of cases using conventional methods (e.g. culture or rapid fluorescent-antibody tests) for detection [7,8]. However, with new molecular diagnostic techniques (e.g. PCR), reports of viral aetiology for acute asthma exacerbation have dramatically increased [8]. The virus types that exacerbate asthma are diverse, which may include influenza A (Flu A), influenza B (Flu B), parainfluenza viruses, respiratory syncytial virus (RSV) and adenovirus (AV) [9,10]. Increased asthmatic rates have been reported with viral co-infections with another respiratory virus than infection with a single respiratory virus [11].

Besides viruses, bacteria also play a substantial role in asthma exacerbation, particularly atypical bacteria such as *Chlamydia pneumoniae* and *M. pneumoniae* [8]. Though typical bacteria are identified in routine microbiology laboratories, *M. pneumoniae* has yet to be identified due to its fastidious growth conditions, which require special culture conditions [12,15]. Therefore, the organism has not been easily identified and escapes in routine bacteriological diagnosis.

To date, there is no data on pathogens responsible, either viral or bacterial, for children with EOA in Sri Lanka, but antibiotics are prescribed widely. It is essential to prescribe antibiotics rationally, targeting specific bacteria to minimize the development of antibiotic resistance. Even though the facility for detecting typical bacteria is available in routine microbiological diagnostic services in Sri Lanka, *M. pneumoniae* is not detected for the above reasons, and molecular diagnostics are expensive. However, macrolides are prescribed widely in EOA, primarily aiming at *M. pneumoniae*. Nevertheless, no macrolide resistance detection methods are available in any microbiology diagnostic services in Sri Lanka. Moreover, molecular typing of *M. pneumoniae* is important epidemiologically to determine circulating macrolide-resistant variants of *M. pneumoniae*, which is also not available in Sri Lanka. Therefore, besides the detection of common respiratory pathogens causing EOA, particular interest was given to the molecular detection of *M. pneumoniae* while performing further molecular studies on the detection of macrolide resistance and multilocus sequence typing (MLST) of the organism.

This study aimed to identify viruses and bacteria associated with EOA, including molecular studies on *M. pneumoniae*, viz., identification, detection of macrolide resistance and typing.

## Methods

Patients and specimen collections were as follows: A study was conducted at the Colombo North Teaching Hospital, Sri Lanka (CNTH-SL), Department of Medical Microbiology, Faculty of Medicine, Ragama, Sri Lanka, and The United Kingdom Health Security, Colindale, UK. The study group comprised 100 children who were 5–14 years of age and were admitted to the paediatric ward with acute EOA, which a consultant paediatrician confirmed. Children with chronic lung diseases or life-threatening asthma were excluded. The second group–those with stable asthma (SA)–included 100 children with a history of bronchial asthma but with no acute exacerbations during the past 4 months who were admitted for other medical conditions or asthmatic children being followed up in the paediatric clinic, CNTH-SL. The sample size was calculated to have an odds ratio of four, alpha risk of 5 % and power of 80 %. The children were recruited from February 2018 to January 2019. Informed written consent was obtained from each child’s parent or guardian. The patient and parent/guardian were interviewed for demographic and clinical data, which were recorded using a pretested, validated questionnaire. Following an explanation of collecting a good quality sputum sample from the child and the parent/guardian, sputum samples were collected into wide-mouthed, screw-capped and sterile containers. In contrast, throat swabs were collected from those who did not produce sputum, adhering to standard precautions using cotton-tipped and long-shaft gel swabs with disposable, sterile tongue depressors. The respiratory samples were placed into universal transport media and transported in ice to the microbiology laboratory within 2 h of collection. The samples were stored at −700 ºC until used for laboratory tests. The data were analysed using R programming language, and a statistically significant level was considered as *P*<0.05.

### Virus detection

The D3 Ultra DFA (direct fluorescent antibody) Respiratory Virus Screening and Identification Kit from Quidel–DiagnosticsHybrids, USA (catalogue number: 01–010000.v2) was used for the qualitative detection of seven common respiratory viruses, viz., Flu A, Flu A, RSV, AV, parainfluenza-1 (PIN-1), parainfluenza-2 (PIN-2) and parainfluenza-3 (PIN-3) viruses. Tests were performed following the manufacturer’s instructions.

### Detection of *M. pneumoniae* DNA by real-time PCR

Sputum and throat swab samples were processed for DNA extraction using QIAamp DNA mini kits by Promega. *M. pneumoniae* DNA detection was performed using a commercially available *M. pneumoniae* real-time PCR (RT-PCR) Kit, GeneProof, Czech Republic (catalogue number: MP/ISEX/025), which targets the DNA sequence encoding CARDS (community-acquired respiratory distress syndrome) toxin. The presence of *M. pneumoniae* DNA was indicated by the fluorescein amidites fluorophore fluorescence. Ready-to-use MasterMix contained uracil-DNA-glycosylase (UDG), minimizing contamination with PCR amplification products. The tests were performed with positive and negative controls originating from the DNA extraction step. The positive control was used for the National Collection of Type Cultures 10 119 *M*. *pneumoniae* strain from the UK Health Security Agency culture collection.

### Detection of macrolide resistance in *M. pneumoniae* by conventional PCR

The *M. pneumoniae* RT-PCR-positive respiratory sample was tested for macrolide resistance with conventional PCR followed by nucleic acid sequence analysis. The standard operating procedure developed at Public Health England for detecting macrolide resistance was followed. In brief, a conventional PCR was performed to amplify the complete region of the potential mutations that confer macrolide resistance (2063, 2064, 2067 and 2617 sites in domain V of 23S rRNA of *M. pneumoniae*). The region of interest was amplified as one product (amplicon size–720 bp) to evaluate potential mutations. The technical details for detecting macrolide resistance are available in Supplement 1 at https://zenodo.org/doi/10.5281/zenodo.10574394.

### Molecular typing of *M. pneumoniae* by MLST

Genotyping of the *M. pneumoniae* isolate was performed by nested MLST in which eight housekeeping genes (*ppa*, *pgm*, *gyrB*, *gmk*, *glyA*, *atpA*, *arcC* and *adk*) were tested with the nested PCR. In brief, a 5 µL volume of extracted DNA from the positive sample was mixed with 4 µL of PCR-grade water and 1 µL of 10 pmol/µL N1 primer mix. Ten microlitres of MasterMix solution (GoTaq® Hot Start Green MasterMix, 100 reactions, Promega, USA, catalogue number: M5122) was added per run as the first PCR in the nested PCR. The second (nested) PCR was run using the N2 primers from the amplicon mix obtained from the first PCR. The exact compositions were used to prepare the PCR mixture, with the only difference being that the N2 primer mix was used instead of the N1 primer mix. Both PCRs included positive controls [genomic DNA of *M. pneumoniae* (Minerva labs, supplier CAMBIO UK, 52-0119)] and negative control [PCR-grade water: water, nuclease-free, 50 mL (2×25 mL) from Promega, USA, catalogue number: P1193]. The products of the second PCR were separated using gel electrophoresis. Once the positive bands for the eight housekeeping genes were confirmed and obtained, the PCR products were sequenced and analysed using BioNumerics to assign alleles and sequence types. The technical details of MLST are available in Supplement 2 at https://zenodo.org/doi/10.5281/zenodo.10574437.

### Bacterial culture

Specimens were tested in standard microbiology culture media for respiratory bacteria.

## Results

### Age, gender and geographical identity

The mean age of the children with EOA and the SA (controls) were 9 years and 9.5 years, respectively. In the children with EOA, 49 % (49/100) were males and 51 % were females, and in children with SA, 47 % were males and 53 % (53/100) were females. There was no statistically significant difference in age, gender and geographical location between the two groups. The patients were clustered geographically in the Gampaha District (western province), 16 km north of Colombo, the capital of Sri Lanka. It is located 20 m (65 ft) above sea level and has a tropical rainforest climate. The district has average annual temperatures of 28 °C (82 °F), which is 0.4 % higher than the average for Sri Lanka, and experiences 269 wet days (74 % p.a.) with approximately 318 mm (12 in.) of precipitation yearly [16].

### Prescription of antibiotics and evidence of infections in children with exacerbation of asthma

**Fig. 1.**
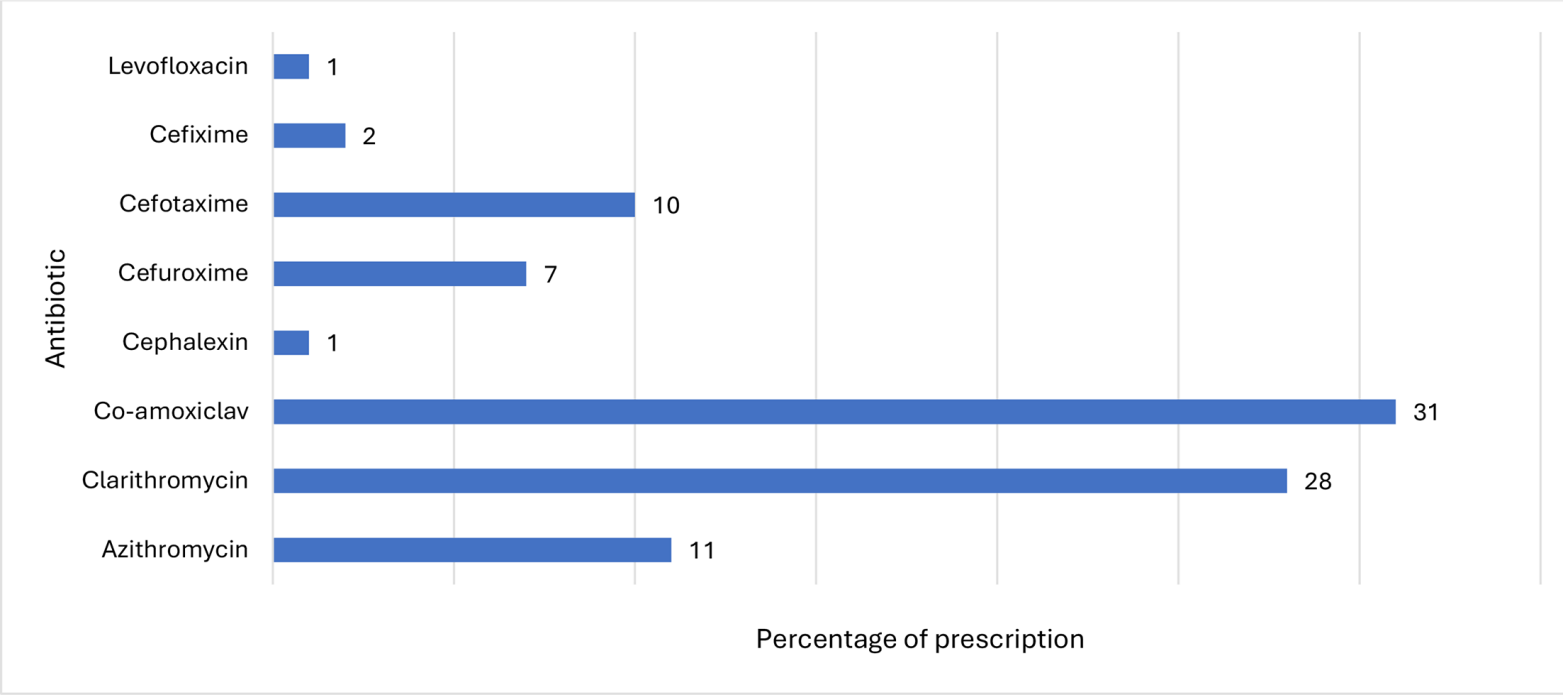
Types of antibiotics in relation to prescription (%) in children with exacerbation of asthma.

In the EOA group, 66 % (66/100) were empirically treated with antibiotics. The antibiotics used are listed in Fig. 1, and 21/100 patients received more than one antibiotic. Macrolides were used to treat 39 % (39/100) of children with EOA; however, none had microbiological evidence of respiratory infection by routine bacteria culture and Gram staining. Fifty-two percent (52/100) of the patients were on regular inhalers, and 42 %(42/100) used inhaled corticosteroids. Fever was evident only in 47 % (47/100) of children with EOA, and only 7 % (7/100) had a high-grade fever (≥1020 F). The haematological profiles of the patients showed that 29.2 % (19/65) had neutrophil leukocytosis (white blood cells >11×103 with >80 % of neutrophil). C-reactive protein values (mg/dL) <10, 10–60, and >60 were 46 % (29/63), 39.7 % (25/63) and 14.3 % (9/63), respectively.

Macrolides (clarithromycin and azithromycin) were used to treat children with EOA 39 % (39/100), while co-amoxiclav was given to 31 % (31/100) of children with EOA. Altogether, beta-lactams (Augmentin, cephalexin, cefuroxime, cefotaxime and cefixime) were given to about half (51/100) of the children with EOA. Antibiotic combinations were given as beta-lactam and macrolide in 16/100 children with EOA, while a beta-lactam, a macrolide and a quinolone were given to one child (1/100).

### Types of respiratory samples and routine microbiological investigations

Respiratory samples from the children with EOA included 78 % sputum and 22 % throat swabs, while all children with SA provided throat swabs. None of the children with EOA or SA underwent the microbiological diagnosis of respiratory viruses, typical bacterial pathogens, or *M. pneumoniae* using culture, serology or molecular biology as part of routine investigations in the hospital. The findings below are the results of the present research.

AV was the most common virus identified (9 %, 9/100) by the DFA respiratory virus screening test. This was significantly higher in children with EOA compared to SA. There were four patients who had dual viral infections. Of the dual positives, two patients had both AV and PIN-3 coinfection, one patient had AV and Flu A, and another patient had Flu AB and RSV coinfection. Routine bacteria culture detected normal oral flora in the respiratory tract in both groups. *M. pneumoniae* was detected by PCR in one patient with exacerbation of asthma (1/100). The latter patient was also positive for Flu B as well.

**Fig. 2.**
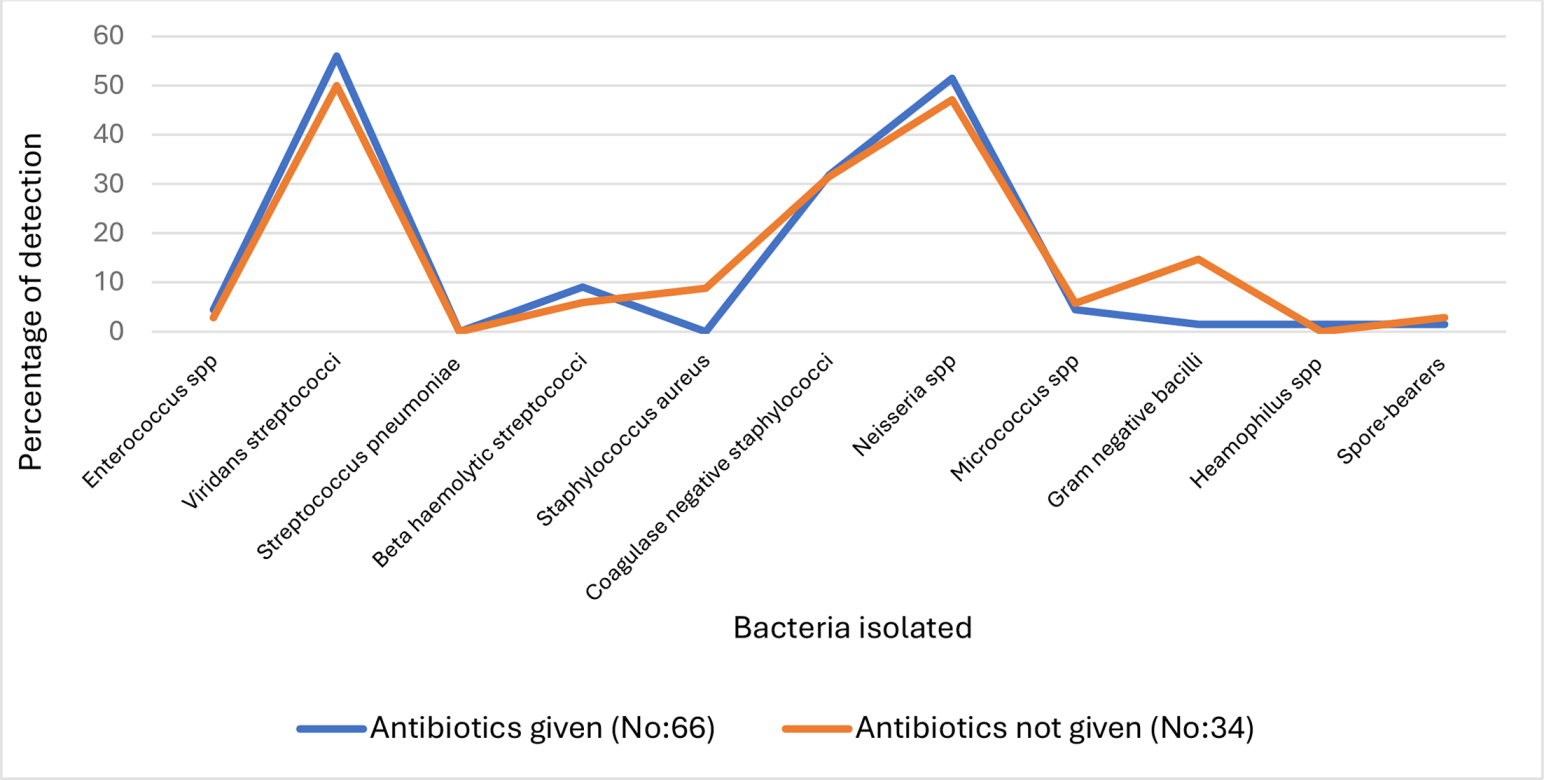
Results of routine bacteriology culture: antibiotics given versus not given.

Fig. 2 depicts recovery bacteria in routine culture in children with EOA in two categories: children who received antibiotics (antibiotics given—No: 66) and children who did not receive antibiotics (antibiotics not given—No: 66). Those who did not receive antibiotics showed a significantly higher isolation rate of *Staphylococcus aureus* (*P*=0.002) and Gram-negative bacilli (*P*=0.003), but the number of patients was very low as 3/34 and 5/34, respectively.

### Detection of macrolide resistance in *M. pneumoniae*

According to the sequence analysis of the products of *M. pneumoniae*-macrolide resistance detection PCR, the *M. pneumoniae* detected from the patient had no mutations that confer macrolide resistance (2063, 2064, 2067 and 2617 sites in domain V of 23S rRNA of *M. pneumoniae*) and confirmed that the sample was macrolide-sensitive.

**Fig. 3.**
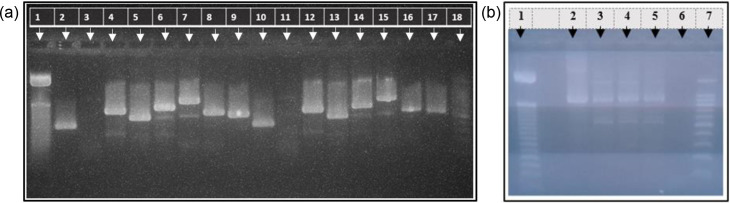
MLST of *M. pneumoniae*. (a) Lane 1—DNA ladder (50 bp), lanes 2 to 9*—M. pneumoniae*-positive control (as per MLST gene order: *ppa*, *pgm*, *gyrB*, *gmk*, *glyA*, *atpA*, *arcC* and *adk*), lanes 10 to 17*—M. pneumoniae*-positive patient sample (as per MLST gene order) and lane 18—DNA ladder (100 bp). Similarly, as in Fig. 3, arrows are indicated to show the highest and lowest DNA ladder size, with one or two additional arrows indicating other DNA bp sizes. Lane 6—negative control. (b) Lanes 1 and 7 show the DNA ladders for the 50 bp and 100 bp, respectively. Lane 2 is positive for *M. pneumoniae* DNA from the positive control sample, and lanes 3, 4 and 5 replicate the *M. pneumoniae*-positive sample from the patient.

Fig. 3(a) shows the gel image of the PCR products from the nested PCR for the MLST of *M. pneumoniae* (positive control and the patient sample), in which eight housekeeping genes were tested and were positive, but the *pgm* gene was not detected. The PCR for the *pgm* gene was repeated, as seen in Fig. 3(b), in which a positive band is seen along with *M. pneumoniae*-positive control and positive patient sample.

The sequence analysis of PCR products of the eight housekeeping genes of the *M. pneumoniae*-positive patient sample was done at the UK Health Security Agency, and the isolate’s sequence type (ST) was reported as ST14.

## Discussion

There was a significantly higher detection rate of adenovirus in the children with EOA compared to those with SA (*P*=0.040) (Table 1). Even though the number of positives for adenovirus was low, 9 % (9/100), it has been previously identified as a significant contributing factor in EOA. According to Tan *et al*., adenovirus has been reported as the second most common cause of near-fatal asthma [24 % (4/17)] [17]. Most strikingly, Aydin *et al*. have demonstrated that sensitivity to house dust mites (HDMs) has the propensity to cause adenovirus infection-induced asthma exacerbations [18]. The authors speculate that the upregulation of adenovirus receptors following inhalation increases the risk of adenovirus infection [18]. The inhabitants of HDM have been recognised in tropical humid environments, similar to the environmental conditions where the present study cohort is located in Sri Lanka [19]. However, further studies are warranted to establish the link between exposure to HDM and the occurrence of respiratory tract infections due to adenovirus in the local setting. No significant difference was detected between children with EOA and those with SA in detecting other respiratory viruses such as Flu A, Flu B, PIN 1–3 and respiratory syncytial virus (Table 1). This is mainly attributable to asymptomatic viral infection in children with SA. According to Galanti *et al*., over 70 % of viral upper respiratory tract infections are asymptomatic [20].

**Table 1.** Detection of respiratory viruses and bacteria

Respiratory virus				Typical bacteria				*M. pneumoniae*		
**Name**	**Children with**		***P* values**	**Name**	**Children with**		**Significance level (*P***)	**Children with**		***P* values**)
	**EOA/100**	**SA/100**			**EOA/89**	**SA/57**		**EOA/100**	**SA/100**	
AV	9	2	**0.040**	VS	53	28	0.505	1	0	0.316
Flu A	1	1	1.000	SPN	0	–	–			
Flu B	2	1	0.567	BHS	05	21	**0.000**			
PIN-1	1	1	1.000	STA	02	02	0.658			
PIN-2	1	2	0.567	CONS	29	13	0.339			
PIN-3	3	2	0.659	ns	50	25	0.406			
RSV	3	2	0.659	GNB	08	03	0.439			

AVadenovirusBHSbeta hemolytic streptococciCONScoagulase-negative staphylococciFlu Ainfluenza AFlu Binfluenza BGNBGram-negative bacilliNScommensal *Neisseria* spp.PIN-1parainfluenzaPIN-2parainfluenza-2PIN-3parainfluenza-3RSVrespiratory syncytial virusSPN*Streptococcus pneumoniae*STA*Staphylococcus aureus*VS*Viridans streptococci*

Notably, antibiotics were used in 66 % of patients, while macrolides were the second most commonly used antibiotic (39 %) (Fig. 1). However, the Global Initiative for Asthma reports that antibiotics are not recommended in EOA [21]. Nevertheless, the inappropriate use of antibiotics in EOA is a significant global concern that substantially impacts patient and public health controls. Baan *et al*. highlighted that antibiotic use was higher in asthmatic compared to non-asthmatic children [22]. In the present study population, 47 % (47/100) had a fever (ranging from 99°F to 104°F), but only 7 % had a high-grade fever (≥102°F), suggestive of bacterial infections.

Routine bacteriology studies in this patient cohort showed an 11.2 % (10/89) rate of bacterial respiratory infections in children with EOA compared to 8.8 % (5/57) in children with SA (Table 1). The low number (10) of pathogenic bacteria in children with EOA may likely be due to the prior use of antibiotics in the prescribed patients. The comparison between those who received antibiotics and those who did not show any significant difference with the isolation of common bacteria (e.g. *S. pneumoniae*, *Streptococcus pyogenes*, *Haemophilus* spp) between the two groups is shown in Fig. 2.

The elevated CRP values of >60 mg dL^−1^ suggest bacterial infection [23]. In the present study, elevated CRP values of >60 mg dL^−1^ were seen in only 14.3 % (9/63) of children with EOA. In the children with EOA who received antibiotics, neutrophil leukocytosis was evident in 29.2 % (19/65). Neutrophil leukocytosis is evident in bacterial infections. Besides, other contributory factors for neutrophil leukocytosis, such as corticosteroid therapy, initial phase of viral infections and hypoxia, may likely contribute to neutrophil leukocytosis in this patient cohort with EOA [24].

Hence, amid no robust haematological or microbiological evidence of bacterial infection in most children with EOA, the high percentage (66 %) of antibiotic prescriptions would need to be revisited. The misuse of antibiotics in upper respiratory tract infections is a substantial challenge in antibiotic stewardship programmes globally and locally [25]. Besides, antibiotic misuse substantially and unnecessarily burdens the country’s health budget. According to the National Health Bulletin of Sri Lanka, the government health expenditure in 2020 was 250 813 million Sri Lankan Rupees, which was 5.6 % (250 813/national expenditure) [26]. Further, it has been shown that 12.7 % of government health expenditure was attributed to antibiotic expenditures [27].

The empirical use of macrolides in this patient cohort, possibly to treat *M. pneumoniae* infection, did not follow microbiological confirmation by serological or bacteriological investigations—probably due to a lack of resources. According to studies worldwide, *M. pneumoniae* has become a trigger for asthma exacerbation in varying percentages from 3.3 to 50 % [28]. However, these assumptions have originated from *M. pneumoniae* antibody tests, which likely represent recent or past infections [28]. This is evident in a study from India in which *M. pneumoniae* DNA detection rate was 6 % (3/50), and specific IgM antibody levels were reported in 42 % (21/50) of children with acute asthma [29]. This serological finding suggests that most are unlikely to have the organism in the respiratory tract. The present study used molecular diagnostics to detect *M. pneumoniae* DNA instead of specific serum antibodies. However, differences in the detection rate in *M. pneumoniae* PCR likely depend on the specific genetic sequence targeted.

In the above study from India, though *M. pneumoniae* DNA detection was 6 % (3/50) in children with EOA, the target sequence used in the PCR was the gene code for the P1 adhesin protein. The target sequence used in the present study was the gene codes for CARDS toxin, considered the most significant pathogenic determinant of *M. pneumoniae* infection [30]. Even though antibiotic use may be postulated for the 1 % of *M. pneumoniae* detection in the current study, molecular detection of *M. pneumoniae* DNA has proven positive even after antibiotic therapy, as PCR can detect specific DNA in viable and non-viable organisms [31]. *M. pneumoniae* infection may not be prevalent or seasonal but may cause specific local outbreaks [32]. However, as reported by Wei *et al*., it has been suggested that individuals on inhaled corticosteroids are less likely to develop EOA following *M. pneumoniae* infection than those not [33]. Accordingly, there is a possible chance of missing those with EOA due to *M. pneumoniae*, as 42 % (42/100) of the children in the present study were on regular corticosteroid inhalers. However, among the 58 children in the EOA group who were not on regular corticosteroid inhalers, only one was positive for *M. pneumoniae* DNA. Hence, the very low 1 % of *M. pneumoniae* infection in the present study cohort would unlikely be attributed to regular corticosteroid inhalers.

A different study done in Sri Lanka on *M. pneumoniae* as a causative agent for acute pharyngitis in adults found that there was a low percentage of 1.5 % (2/138), highlighting the scarcity of *M. pneumoniae* in upper respiratory tract infections, which is a known cause of exacerbation of asthma [34]. However, the previous study involved adults whose asthma signs and symptoms were not noted, in contrast with the present study, which involved children with asthma.

The 1 % of *M. pneumoniae* infection in children with EOA implies that *M. pneumoniae* had no significant role in this study cohort (Table 1). However, there is a substantial disparity between the empiric use of macrolides for *M. pneumoniae* [39 % (39/100)] and laboratory evidence of its presence in one patient with EOA (Table 1). As is well acknowledged, the use of antibiotics without proven evidence of bacterial respiratory infection promotes the development of resistance against other bacteria, predominantly * S. pneumoniae* residing in the upper respiratory tract as a commensal, which is a common cause of community-acquired pneumonia [35]. Studies have shown that macrolide’s wide/inappropriate use is associated with <10 % to >90 % macrolide resistance in *S. pneumoniae* [36,37]. According to a study in Sri Lanka as part of a global *S. pneumoniae* research network in 2005–2007, erythromycin resistance in *S. pneumoniae* in Sri Lanka was 60.7 % [38]. This has increased to 82.1 % (147/179) in hospitalized children with respiratory symptoms [39]. The wide use of macrolides is of substantial concern, with reported resistance of such a significant magnitude [40]. Besides being an antibiotic, macrolides have anti-inflammatory properties, which may be inappropriately prescribed for EOA [41]. Hence, the empiric use of antibiotics in children with asthma may be better targeted with respiratory pathogen screening to inform treatment.

It has been reported that macrolide-resistant *M. pneumoniae* (MRMP) has emerged due to the frequent usage of macrolides [42]. The prevalence of MRMP ranges from 2 to 26 % in European countries and 30 % in Israel [43,45]. It is much higher in Asia, where this first emerged in 2000, and now exceeds 90 % in some areas of China and Japan [46,47]. Although the *M. pneumoniae* detected in the present study was macrolide-sensitive, the result was limited to a single positive sample in children with EOA. However, establishing the testing protocol for detecting macrolide resistance in *M. pneumoniae* for the first time in Sri Lanka has made the facility available for future studies. This would facilitate the evaluation of a substantial number of *M. pneumoniae* isolates for better representation of macrolide resistance in the country. A previous study by Wijesooriya *et al*. showed that the prevalence of *M. pneumoniae* in patients with community-acquired pneumonia in Sri Lanka was 17.5 % (17/97) [34]. *M. pneumoniae* infection has been reported to occur more often as specific outbreaks, unlike *S. pneumoniae*, which is prevalent in the community [48,49]. This would be possible because of the low rate (1/100) of *M. pneumoniae* in the present study, conducted during a non-epidemic period with this infection.

The typing of *M. pneumoniae* was done by genetic characterization of the organism via MLST, which is essential in the analysis of epidemiological relationships. To date, 46 MLST types of *M. pneumoniae* have been identified globally [50]. The *M. pneumoniae* sequence type 3 (ST3) has been reported as the most prevalent [51]. The present study reports the prototype characterization of *M. pneumoniae* isolated from Sri Lanka. Accordingly, the first *M. pneumoniae* typed from Sri Lanka was ST14. *M. pneumoniae* ST14 has been reported in Japan, South Korea, France and the USA [52]. Further, there is a link between macrolide resistance and specific ST types; in regions where MRMP is high, the clonal expansion of ST3 has been noted [53]. Further, ST17 has also been recorded as a small MRMP clone from Taiwan [54]. Nevertheless, MRMP has been detected within other ST types in different frequencies, including ST14 [52]. Yet, the *M. pneumoniae* ST14 identified in the present study was macrolide-sensitive. However, establishing the typing of *M. pneumoniae* for the first time in Sri Lanka has made the facility available for future studies. This would facilitate the evaluation of *M. pneumoniae* isolates and support monitoring macrolide resistance in the country.

## Conclusion

Adenovirus infection was essential for acute asthma exacerbation in this study cohort. There was low evidence (5/89) of typical bacterial infection and one case of *M. pneumoniae* infection (1/100) associated with asthma exacerbation. The *M. pneumoniae* was identified as ST14 by MLST, and its macrolide sensitivity was the first characterization in Sri Lanka. Antibiotics, including beta-lactams and macrolides, have empirically been used widely in children for respiratory tract infections. There is a substantial disparity between the use of antibiotics and evidence of bacterial aetiology. Hence, the empiric use of antibiotics in children with asthma may better be targeted with respiratory pathogen screening to inform appropriate treatment.

This small study of 200 selected patients (100 with EOA and 100 with SA) has provided initial findings regarding the relationship between EOA, infectious agents and inappropriate antibiotic prescriptions in Sri Lanka, which has a reported high rate of a/b-resistant bacteria [55]. Further and expanded studies with a larger statistically valid population would provide more significant findings to help reduce antibiotic resistance and its inappropriate use.

## Abbreviations

AV: adenovirus
BHS: beta hemolytic streptococci
CARDS: community-acquired respiratory distress syndrome
CONS: coagulase-negative staphylococci
DFA: direct fluorescent antibody
EOA: exacerbation of bronchial asthma
Flu: influenza
GNB: gram-negative bacilli
HDM: house dust mite
MAb: monoclonal antibody
MLST: multilocus sequence typing
MRMP: macrolide-resistant *M. pneumoniae*
NS: commensal *Neisseria* spp.
PIN: parainfluenza
RI: respiratory infection
RSV: respiratory syncytial virus
RT-PCR: real-time PCR
SA: stable asthma
SPN: *Streptococcus pneumoniae*
ST: sequence type
STA: *Staphylococcus aureus*
UDG: uracil-DNA-glycosylase
VS: Viridans streptococci
